# A novel pre-fusion conformation-specific neutralizing epitope on the respiratory syncytial virus fusion protein

**DOI:** 10.1038/nmicrobiol.2016.271

**Published:** 2017-01-30

**Authors:** Jarrod J. Mousa, Nurgun Kose, Pranathi Matta, Pavlo Gilchuk, James E. Crowe

**Affiliations:** 1 Vanderbilt Vaccine Center, Vanderbilt University Medical Center, Nashville, Tennessee 37232, USA; 2 Department of Pathology, Microbiology and Immunology, Vanderbilt University, Nashville, Tennessee 37232, USA; 3 Department of Pediatrics, Vanderbilt University School of Medicine, Nashville, Tennessee 37232, USA

## Abstract

Respiratory syncytial virus (RSV) remains a major human pathogen, infecting the majority of infants before age two and causing re-infection throughout life. Despite decades of RSV research, there is no licensed RSV vaccine. Most candidate vaccines studied to date have incorporated the RSV fusion (F) surface glycoprotein, because the sequence of F is highly conserved among strains of RSV. To better define the human B cell response to RSV F, we isolated from a single donor 13 new neutralizing human monoclonal antibodies (mAbs) that recognize the RSV F protein in the pre-fusion conformation. Epitope binning studies showed that the majority of neutralizing mAbs targeted a new antigenic site on the globular head domain of F, designated here antigenic site VIII, which occupies an intermediate position between the previously defined major antigenic sites II and site Ø. Antibodies to site VIII competed for binding with antibodies to both of those adjacent neutralizing sites. The new mAbs exhibited unusual breadth for pre-fusion F-specific antibodies, cross-reacting with F proteins from both RSV subgroups A and B viruses. We solved the X-ray crystal structure of one site VIII mAb, hRSV90, in complex with pre-fusion RSV F protein. The structure revealed a large footprint of interaction for hRSV90 on RSV F, in which the heavy chain and light chain both have specific interactions mediating binding to site VIII, the heavy chain overlaps with site Ø, and the light chain interacts partially with site II.

RSV expresses three surface proteins: attachment (G), small hydrophobic (SH) and fusion (F) proteins. The G and F glyco-proteins are the targets of neutralizing antibodies. Although the RSV G protein does induce neutralizing antibodies, antigenic diversity in G proteins among RSV strains makes it difficult to design a broadly protective vaccine candidate based on immunogenicity to this protein. Although there is no licensed RSV vaccine, a prophylactic monoclonal antibody (mAb), palivizumab^1^ (Synagis; MedImmune), is available for prophylactic treatment of high-risk infants, yet the high cost and moderate efficacy limit its use. The F protein is a class I fusion glycoprotein that adopts two conformations during viral infection. The pre-fusion F conformation is metastable and is triggered easily to the post-fusion conformation, resulting in a dramatic change involving the formation of a six-helix bundle extending the hydrophobic fusion peptide into the host cell membrane^2^. Recent structural breakthroughs in X-ray crystallography have provided atomic-resolution detail of the post-fusion and pre-fusion F conformations^3,4^. Furthermore, structure-based design of the F protein has resulted in stabilized F constructs (Ds-Cav1 and SC-TM) that retain components of the pre-fusion F conformation and induce neutralizing antibody immune responses^5,6^. Several neutralizing antigenic sites have been reported previously, recognized by the representative mAbs 131–2a (ref. 7) (site I), palivizumab^1^ and motavizumab^8^ (site II), 101F (ref. 9) (site IV), 7.936 (ref. 10 (site V, near amino acid 447), 7.916 and 9.432 (ref. 10) (site VI, near amino acid 432) and the recently discovered pre-fusion specific mAb D25 (ref. 4) (site Ø). Furthermore, a quaternary-dependent pre-fusion-specific epitope has been described using mAb AM14 (ref. 11), as well as an RSV/metapneumovirus cross-neutralizing region near site II (ref. 12). Antigenic sites II and IV–VI are retained in both the pre- and post-fusion conformations of F (ref. 13), as evidenced by the X-ray structures having exposed epitopes at these sites in both conformations. Antigenic site Ø is pre-fusion-specific, as the conformational epitope is lost in the rearrangement to the post-fusion conformation.

We recently described the isolation and characterization of several new human mAbs targeting antigenic sites I and II, which were identified by screening for binding to the RSV strain A2 F protein in the post-fusion conformation^14^. Several site II mAbs were described that are potently neutralizing, including clones with binding poses on site II that differ from that of palivizumab and exhibit distinct functional patterns. While site II is the target of palivizumab and the second-generation mAb motavizumab, and has been shown to induce potently neutralizing mAbs, antigenic site II may not be the optimal antigenic site to induce protective mAbs against RSV infection. Non-neutralizing mAbs that recognize a nearby newly recognized antigenic site (site VII, centred near amino acid Leu467) compete for binding at antigenic site II, particularly in the context of the post-fusion conformation^14^. Recent experiments suggested a dominant role for epitopes in the pre-fusion conformation of RSV F in the induction of serum neutralizing antibodies, particularly a major role for antigenic site Ø in immunogenicity^15^. However, although site Ø-specific mAbs are indeed among the most potently neutralizing, very few human mAbs to this site have been isolated and characterized. To further characterize the human immune response to the RSV F protein, and in particular the pre-fusion form of RSV F, we used hybridoma technology^16^ to isolate new mAbs to RSV F and identified 13 new neutralizing human mAbs that recognize the pre-fusion conformation of the RSV F protein.

## Human antibody generation

Peripheral blood mononuclear cells were isolated from a single eight-year-old human donor by Ficoll-gradient centrifugation and the cells were frozen for later use. For B cell screening, thawed cells were transformed with Epstein–Barr virus and plated in 384-well plates to generate immortalized lymphoblastoid cell lines. Supernatants from the transformed cells were screened for antibodies binding to a highly stable pre-fusion conformation of RSV A2 F, using the single-chain triple mutant (SC-TM) construct^6^. B cells from cultures producing antibodies reactive with the pre-fusion F protein were electrofused with the HMMA2.5 myeloma cell line to generate stable hybridoma cell lines. To obtain homogeneous antibody secretions, hybridoma cells were cloned biologically by single-cell flow cytometric sorting. Hybridomas were expanded stepwise to 1 l cultures and mAbs were purified from filtered culture supernatants. Purified mAb yields from the cultures ranged widely depending on the hybridoma clone, with the lowest being 1.5 mg l^−1^ and the highest nearing 30 mg l^−1^ (Table 1). RSVF-specific mAbs were characterized by antibody isotyping analysis. All mAbs except hRSV130 were of the immunoglobulin G1 (IgG1) subclass, and the majority of light chains were of the kappa subtype (Table 1).

## Antibody neutralization and binding specificity

To determine neutralizing potency, we tested the mAbs by plaque-reduction assay using RSV A2 (subgroup A virus), RSV 18537 B (subgroup B virus) and RSV Long (subgroup A virus). All mAbs had half-maximal inhibitory concentration (IC_50_) values less than 1 μg ml^−1^ for RSV A2(Table 1 and Supplementary Fig. 1). All mAbs also neutralized RSV 18537 B and RSV Long strains, indicating the mAbs have neutralizing breadth across RSV A and B subgroups. We determined the half-maximal effective concentration for binding (EC_50_) values by enzyme-linked immunosorbent assay to test the F-protein strain specificity and preference for pre-fusion versus post-fusion F conformations. We used four different proteins in the binding experiments: RSV strain A2 pre-fusion F (SC-TM), RSV strain 18537 B pre-fusion F (Ds-Cav1), RSV strain A2 post-fusion F and RSV strain 18537 post-fusion F (Table 1 and Supplementary Fig. 2). Interestingly, all mAbs except hRSV131 and hRSV12 bound specifically to the pre-fusion conformation of F, with hRSV131 and hRSV12 binding both pre-fusion and post-fusion conformations from RSV strain A2 or 18537 B. Furthermore, the mAbs all exhibited cross-reactive binding to F of both RSV subgroups A and B. Cross-reactive pre-fusion conformation-specific mAbs have been reported only in one case, that of the quaternary-epitope dependent mAb AM14 (ref. 11). Other pre-fusion specific mAbs, such as D25, bind at antigenic site Ø, yet these mAbs are specific for RSV subgroup A. The mAbs hRSV90 and hRSV20 were particularly potent in neutralizing RSV strain A2, with IC_50_ values <45 ng ml^−1^. The remaining mAbs had neutralizing IC_50_ values at or less than 350 ng ml^−1^, a level of activity that is similar to, or better than that of, the licensed mAb palivizumab^1^. Binding EC_50_ values were similar for the majority of the neutralizing mAbs, suggesting that the binding pose or fine epitope specificity, rather than the affinity, are the principal determinants of differential neutralizing potency. As the isolated mAbs have therapeutic potential for prophylactic treatment of RSV, we tested the mAbs for self-reactivity using a human cell line (Jurkat). None of the mAbs exhibited significant self-reactivity as compared to a known IGHV4–34*01 self-reactive mAb, or to an antigen-specific mAb control (Supplementary Fig. 3).

## Sequence similarity

The transcribed antibody heavy and light chain variable genes from hybridoma cell lines were sequenced to determine if there were any common genetic features in transcripts encoding these mAbs. We found remarkable genetic similarities among clones, suggesting common structural features deriving from the germline gene-encoded antibody structures. Two genetic clusters were observed among neutralizing mAbs (Table 2). Four neutralizing mAbs (hRSV90, hRSV20, hRSV130 and hRSV97) were encoded by Vh3–9*01. Of these, hRSV90, hRSV20 and hRSV130 used the same Jh gene. Furthermore, hRSV90, hRSV20 and hRSV130 used a similar light chain variable gene Vl3–15*01, and these mAbs have nearly identical light chain junction regions. Although hRSV90 and hRSV20 shared nearly identical gene segment usage, the two mAbs are probably not clonal siblings due to a heavy chain complementarity determining region 3 (HCDR3) insertion in hRSV20.

## Epitope binning

**Figure 1:**
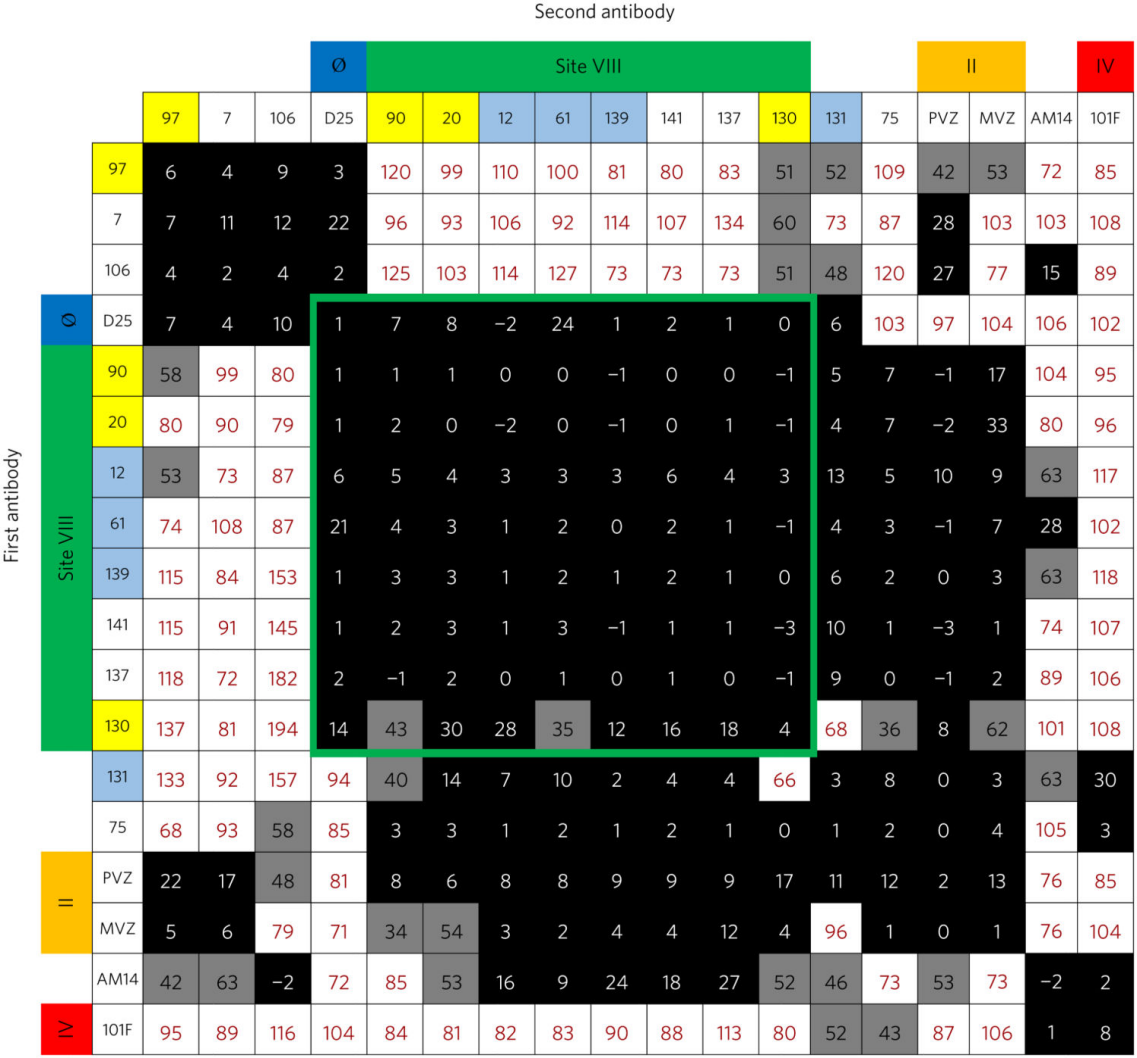
Epitope binning for the RSV F-specific mAbs Data indicate the per cent binding of the second antibody in the presence of the primary antibody, as compared to the second antibody alone. Cells filled in black indicate full competition, in which ≤33% of the uncompeted signal is observed, grey indicates intermediate competition, if the signal is between 33 and 66%, and white indicates non-competing, if the signal is ≥66%. Antigenic sites are highlighted at the top and side based on competition-binding with the control mAbs D25 (site Ø), palivizumab (PVZ) or motavizumab (MVZ) (site II), or 101F (site IV). Those coloured in yellow are encoded by Vh3–9*01, and those in light blue are encoded by Vh1–18*01. Competition for binding with D25 and palivizumab/motavizumab revealed a novel antigenic site VIII bound by antibodies that compete with both mAbs specific for site Ø or mAbs specific for site II. The site VIII competition-binding group is indicated by a green border.

To determine the antigenic sites targeted by the mAbs, we performed epitope binning using biolayer interferometry. RSV A2 F SC-TM protein was loaded onto anti-penta-HIS biosensor tips and then one RSV mAb was loaded onto the F protein. Following this, a second RSV mAb was loaded and competition was measured (Fig. 1). Recombinant forms of mAbs D25, palivizumab and mota-vizumab, and 101F were used as controls in mapping antigenic sites Ø, II and IV, respectively. Additionally, the trimer-dependent mAb AM14 was used in the study. Interestingly, we discovered a unique competition-binding pattern (and by inference a new anti-genic site) for an antibody cluster that competed with both antigenic sites Ø and II, which we designated antigenic site VIII. Antigenic sites Ø and II are over 40 Å apart in the RSV F SC-TM structure (PDB: 5C6B), yet antigenic VIII site evidently possesses residues in or near both sites Ø and II, as competition was observed between site VIII-specific mAbs and palivizumab/motavizumab. Several of the new mAbs also competed with AM14, suggesting binding near the antigenic region for that mAb. It is worth noting that three of the new pre-fusion F-specific mAbs we isolated (designated hRSV97, hRSV7 and hRSV106) showed competition for binding with palivizumab/motavizumab but did not compete with mAbs in the antigenic site VIII competition block. This finding suggests that the mAbs target an alternative antigenic site. The mAbs hRSV75, 131 and AM14 competed for binding with the antigenic site IV mAb 101F in addition to competing for mAbs at site VIII. As expected, hRSV90 and hRSV20 exhibited similar competition-binding patterns, consistent with the identical gene usage among these mAbs. Vh1–18*01 gene-encoded mAbs hRSV12, hRSV61, hRSV139 and hRSV131 also showed similar competition-binding patterns with Vh3–9*01 gene-encoded mAbs hRSV90 and hRSV20. Interestingly, mAbs hRSV131 and hRSV12 shared similar gene usage to hRSV61 and hRSV139, yet the former bind both pre- and post-fusion conformations of F protein, suggesting subtle changes caused by somatic mutations in the differing recombined genes incorporating Vh1–18*01 and/or differing light chain genes altered mAb specificity and created an antigenic site VIII mAb that has both pre- and post-fusion F binding capacity. It is worth noting that hRSV131 showed appreciable binding to the metapneu-movirus fusion protein (Supplementary Fig. 2). This unusual cross-reactive binding pattern, coupled with the epitope binning data, suggest the mAb may bind at a similar location to the previously described human mAb MPE8 (ref. 12).

## X-ray crystal structure of hRSV90-RSV F SC-TM complex

To characterize the newly identified antigenic site VIII further, we determined the X-ray crystal structure of the most potently neutralizing mAb, hRSV90, in complex with pre-fusion RSV A2 F SC-TM protein. hRSV90-Fab was obtained by papain cleavage from the hybridoma-secreted IgG, complexed with pre-fusion F protein and purified by size-exclusion chromatography. The Fab-F complex eluted in one band and crystals formed in several conditions including 1.5 M LiSO_4_·H_2_O/100 mM HEPES pH 7.5 and 20% PEG 550 MME/100 mM NaCl/100 mM BICINE pH 9.0. The largest and best diffracting crystals were obtained in 30% PEG 400/200 mM MgCl_2_·6H_2_O/100 mM HEPES pH 7.5. The diffraction was significantly anisotropic, so the processed data were submitted to the diffraction anisotropy server^17^, which gave *a* = 3.6, *b* = 3.6 and *c* = 3.1. The structure was solved to 3.1 Å (*R*_work_/*R*_free_ = 22/26%) using molecular replacement with the RSV A2 FSC-TM structure(PDB:5C6B) and an alanine truncated Fab structure (PDB: 4Q9Q) as search models (Supplementary Table 1). Density for the Fab was observed at the interface between antigenic sites Ø and II, consistent with the competition-binding data, and identified a novel antigenic site that is the target of potently neutralizing antibodies (Supplementary Fig. 4).

**Figure 2:**
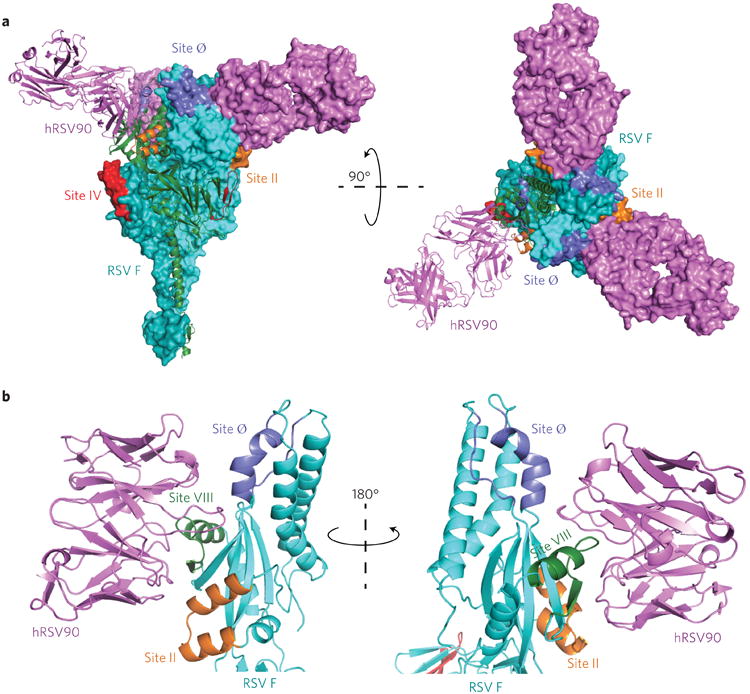
X-ray crystal structure of hRSV90-RSV F SC-TM complex **a**, Overall structure of the hRSV90-RSV F trimer. Antigenic sites are labelled with site IV in red, site II in orange and site Ø in purple. hRSV90-Fab (magenta) binds at a unique site between antigenic sites II and Ø. The structure is turned 90° and shown looking down at the viral membrane. One protomer is shown as a cartoon in each representation, where the RSV F is coloured cyan. **b**, Overall interactions between hRSV90 and pre-fusion RSV F. hRSV90 binds at two clefts around the newly identified antigenic site VIII (green). The heavy chain is close to site Ø (purple), while the light chain is close to site II (orange). The unique features of site VIII comprise residues 163–181 of the RSV F protein. The structure is turned 180° to show both sides of the Fab-RSV F interaction.

The asymmetric unit contained one RSV F protomer and one hRSV90-Fab molecule, and the complex crystallized in a trimer based on the position of symmetry related partners (Fig. 2a). The trimerization domain of the recombinant F protein was clearly visible in the electron density (Supplementary Fig. 4). The three hRSV90-Fabs in the trimer structure are distant from each other and each Fab interacts only with one protomer of F, suggesting hRSV90 is not trimer-specific. hRSV90 is positioned ∼30° upward from the horizontal F axis, engaging only the top 37 Å of the F protein. When viewed from the top face, the three Fabs radiate outward from the RSV F trimer, engaging a 35 Å surface (Fig. 2a). hRSV90 binds to RSV F primarily through the ‘helix-loop-sheet’ motif at residues 163–181 (Fig. 1b). The Fab uses an 18-residue HCDR3 that is bulged at the torso and that inserts itself between antigenic sites Ø and II at the helix-loop-sheet motif. The hRSV90 heavy chain interacts with residues 163–181 and site Ø, while the light chain interacts with residues 163–181 and site II. hRSV90 interacts only with the DIII domain of RSV F (Supplementary Fig. 5a). The helix-loop-sheet residues are rearranged in the post-fusion conformation, forming part of the extended helix of the heptad repeat A portion (Supplementary Fig. 5b). This rearrangement explains the pre-fusion specificity of hRSV90 and similar mAbs described here, as the primary antigenic region is absent in the post-fusion conformation. This property is similar for mAb D25, as the site Ø antigenic region is rearranged in post-fusion RSV F.

**Figure 3:**
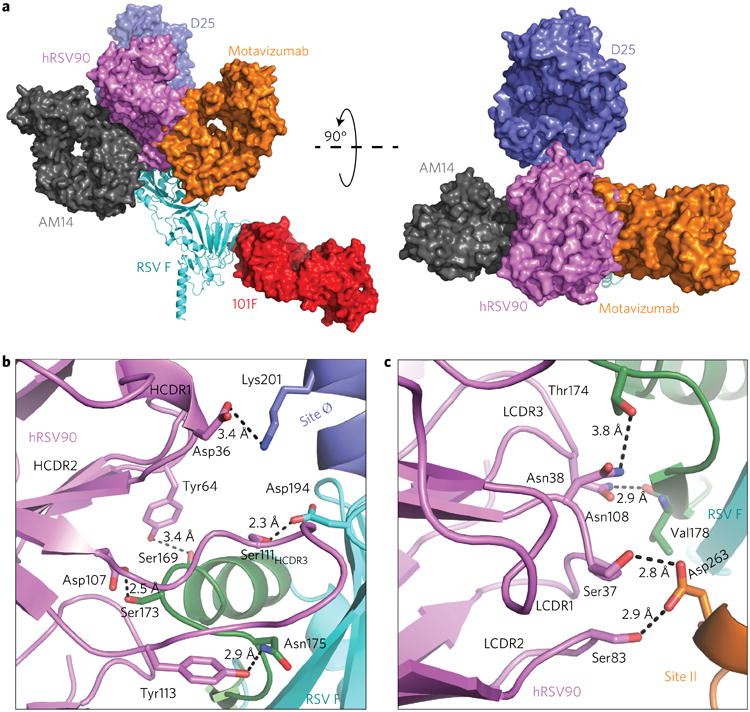
Comparison between hRSV90 and known antigenic sites and interactions between hRSV90 and pre-fusion RSV F **a**, Known X-ray structures are superimposed on the hRSV90-RSV F structure to compare antigenic sites. mAb AM14 (PDB: 4ZYP) and mAb D25 (PDB: 4JHW) were overlaid at RSV F, motavizumab (PDB: 3IXT) was overlaid at the site II peptide, and mAb 101F (PDB: 3O41) was overlaid at the site IV peptide. The structure is turned 90° to show the comparison looking down on the viral membrane. hRSV90 overlaps with mAbs at sites II and Ø. **b**, Specific interactions between the hRSV90 heavy chain and RSV F are shown. hRSV90 is shown in magenta, antigenic site VIII in green, site II in orange and site Ø in purple, while the RSV F protein is coloured cyan. The heavy chain CDR3 mediates binding to site VIII and site Ø. **c**, Interactions between the hRSV90 light chain and RSV F. The colours are as in **b**. The hRSV90 light chain mediates binding to antigenic site VIII and II.

When comparing previously described antigenic sites to site VIII, hRSV90 is nestled between sites II and Ø, and is also in close proximity to the trimer-dependent mAb AM14 site, while being distant from 101F at antigenic site IV. While AM14 and mota-vizumab bind at opposite interfaces of the RSV F protein in an orientation that is approximately parallel, hRSV90 is turned nearly 90°, engaging with RSV F in a perpendicular orientation. When observing the antigenic overlay from the apex, bound hRSV90 sits between bound AM14 and motavizumab on F, while being shifted 80° downward from D25 (Fig. 3a).

hRSV90 uses the heavy chain to interact with antigenic sites VIII and Ø, binding at the interface between the two sites. The HCDR3 sits perpendicular to the long axis of the RSV F protein, on top of site VIII. The torso of the HCDR3 interacts with site VIII through Asp107, hydrogen-bonding to the loop residue Ser173 (Fig. 3b). The HCDR3 contains multiple Tyr residues. However, only Tyr113 is involved in hydrogen bonding, interacting with Asn175, also on the loop portion of antigenic site VIII. Tyr109 and Tyr112.1 are pointed directly toward the RSV F protein, but they do not interact directly with the protein. Possibly, these Tyr residues are involved in indirect bonding through water molecules, but this mode of interaction is unclear in the 3.1 Å resolution structure. We considered whether the Tyr residues were post-translationally modified by sulfation, but we did not observe density for sulfate at the tyrosine residues. HCDR3 Ser111 hydrogen bonds to Asp194 just below site Ø. Additional interactions are present with RSV F through the HCDR2 and HCDR1. The HCDR2 interacts with the helix of site VIII by hydrogen bonding to Ser169 through Tyr64. A single distant interaction with site Ø is mediated by the CDR1 residue Asp36 to RSV F Lys201. While the heavy chain interacts with site VIII and site Ø, the light chain interaction provides the basis for hRSV90 competition with antibodies that recognize antigenic site II (Fig. 3c). Both the light chain CDR2 (LCDR2) and LCDR1 are in sufficiently close proximity to hydrogen-bond with site II residue Asp263 via interactions with Ser83 and Ser37, respectively. A further hydrogen bond from LCDR1 Asn38 allows interaction with the site VIII loop residue Thr174. Furthermore, the single site VIII sheet direct hydrogen bond is provided by LCDR3 Asn108 interacting with the backbone carbonyl of Val178.

## Mutagenesis of hydrogen-bond interactions

To confirm the X-ray structure and determine the critical residues responsible for hRSV90 binding to RSV F, we mutated each of the contact residues observed in the X-ray structure (Table 3 and Supplementary Figs 6–8). Surprisingly, mutating individual residues to alanine showed no significant effect on hRSV90 binding. To probe the interactions further, we mutated each residue to arginine to test for steric effects. All site VIII mAbs identified from epitope binning showed loss of binding for one or more mutants, confirming the site of the binding region for these mAbs. Mutant Ser173Arg (interacting with hRSV90 HCDR3) resulted in loss of binding for hRSV90, hRSV20 and hRSV130. Mutant Thr174Arg (interacting with hRSV90 LCDR3) caused loss of binding for mAbs hRSV90, hRSV20, hRSV130, hRSV61, hRSV137 and hRSV141. The mAbs clustered outside site VIII from epitope binning experiments (hRSV97, hRSV7, hRSV106, hRSV131 and hRSV75) retained binding in all tested mutants, also confirming the uniqueness of antigenic site VIII. We did observe significant loss of binding for mAb D25 when mutating the site Ø residue Lys201Ala, and this binding was rescued in the Lys201Arg mutation. The epitope for AM14 was previously determined using MARM generation, which showed Leu160Ser and Asn183Lys mutations, among others. Asn183 is positioned at the end of the loop of the site VIII epitope and does not interact with hRSV90.

## Discussion

The protein sequence in the site VIII epitope is highly conserved in field isolates between RSV A and B subgroups (Supplementary Fig. 9), similar to antigenic sites II and IV. The X-ray structure of hRSV90 with RSV F defines the structural basis for the newly discovered antigenic site VIII. The most potently neutralizing RSV mAbs previously described include those at antigenic site Ø, a pre-fusion specific epitope. We provide new insight into a novel pre-fusion conformation-specific major antigenic site residing between antigenic sites Ø and II. The isolated mAbs are comparable to the best-in-class RSV antibodies described to date in terms of binding and neutralization. This new antigenic site has probably been unrecognized previously due to the majority of human antibody experiments using polyclonal serum. Indeed, when measuring serum antibody competition-binding to site Ø or site II, the activity of antibodies binding to antigenic site VIII may have been grouped into one or the other sites. However, site VIII contains unique epitopes for potently neutralizing antibodies. Further experimentation will probably identify additional mAbs targeting the site VIII epitope. Site VIII also induces broadly cross-reactive F protein antibodies that recognize both subgroups of RSV. The previously discovered cross-reactive mAb AM14 provides the same cross-reactive response. However, site VIII resides on one protomer. This characteristic of site VIII may prove useful for future structure-based vaccine designs, because highly quaternary sites comprising domains from multiple protomers are difficult to recapitulate with synthetic antigens.

## Methods

### Enzyme linked immunosorbent assay (ELISA)

For recombinant protein capture (ELISA), 384-well plates were incubated with 2 μg ml^−1^ of antigen overnight at 4°C. The plates were blocked for one hour with 2% non-fat dry milk supplemented with 2% goat serum. Plates were washed three times with PBS-T and primary mAbs or hybridoma cell culture supernatants were applied to wells for one hour. Plates were washed with PBS-T four times before applying 25 μl secondary alkaline phosphatase-conjugated antibody (goat anti-human IgG Fc, Meridian Life Science) at a dilution of 1:4,000 in blocking solution. After a 1 h incubation, the plates were washed five times with PBS-T and 25 μl of substrate solution (1 mg ml^−1^ pNPP disodium salt hexahydrate, Sigma) was added to each well. The plates were incubated at room temperature for approximately 30 min before reading the optical density at 405 nm on a Biotek plate reader. Experiments with the RSV F mutants were conducted similarly.

### Human hybridoma generation

The generation of human hybridomas has been described previously^14^. Briefly, PBMCs were isolated from a single human donor and were transformed with Epstein–Barr virus (EBV). After seven to ten days, culture supernatants were screened for binding to recombinant RSV A2 F SC-TM. Cells from positive wells were fused with HMMA2.5 myeloma cells by electrofusion to generate hybridomas^19^. Hybridomas were placed in hypoxanthine-aminopterin-thymidine and ouabain selection media and screened after two weeks for mAb production by ELISA. Cells from wells with reactive supernatants were expanded to 48-well plates for one week before being screened again by ELISA and then subjected to single-cell flow cytometric cell sorting. After cell sorting into 384-well plates containing Medium E (StemCell Technologies), hybridomas were screened by ELISA before expansion into both 48-well and 12-well plates. Hybridoma cells lines were expanded in Medium E until 80% confluent in 75 cm^2^ flasks. For antibody production, cells from one 75 cm^2^ cell culture flask were collected with a cell scraper and expanded to four 225 cm^2^ cell culture flasks in serum-free medium (Hybridoma-SFM, GIBCO). After 21 days, supernatants were filtered using 0.45 μm pore size filter devices.

### Virus sequencing

RSV A2 and RSV Long viruses were sequenced at the region surrounding antigenic site II to confirm strain specificity. Hep-2 cells were infected with each RSV strain and cells were collected after four days. RNA was extracted using Qiagen RNeasy Mini kit and RT–PCR was conducted using Clontech Primescript HIFI Onestep kit following the manufacturer's protocol. The primers used for the RT–PCR and subsequent sequencing were 5′-GTTACCTATTGTGAA CAAGC-3′ and 5′-GCTGCTTACATCTGTTTTTG-3′. No mutations were present in the amplified region, which matched NCBI Sequence ID CUS01855.1 for RSV A2 and NCBI Sequence ID AAC18601.1 for RSV Long strain.

### RSV plaque neutralization experiments

The mAbs isolated from hybridoma supernatants were incubated 1:1 with a suspension of infectious RSV strain A2, 18537 B, or Long for 1 h. Following this, confluent HEp-2 cell culture monolayers, maintained in Opti-MEM I + GlutaMAX (Fisher) supplemented with 2% fetal bovine serum at 37 °C in a CO_2_ incubator, in 24-well plates, were inoculated with 50 μl of the antibody:virus mixture for 1 h. After the hour, cells were overlaid with 1 ml of 0.75% methylcellulose dissolved in Opti-MEM I + GlutaMAX. Cells were incubated for four days, after which the plaques were visualized by fixing cells with 10% neutral-buffered formalin and staining with crystal violet. Plaques were counted and compared to a virus control. Data were analysed with Prism software (GraphPad) to obtain IC_50_ values.

### Human mAb and Fab production and purification

For antibody purification from hybridoma supernates, HiTrap MabSelectSure columns (GE Healthcare Life Sciences) were used to purify antibodies using the manufacturer's protocol. To obtain fragment antigen-binding (Fab) fragments, papain digestion was used (Pierce Fab Preparation Kit, Thermo Scientific). Fab fragments were purified by removing IgG and Fc contaminants using a HiTrap MabSelectSure column followed by purification with an anti-CH1 column (GE Healthcare Life Sciences).

### Assessing self-reactivity of mAbs by flow cytometry

Cultures of Jurkat E6-1 (ATCC) and lentivirus transducted Jurkat E6-1 cells that express Zaire Ebolavirus glycoprotein on the surface (gift from C. Davis and R. Ahmed, Emory University School of Medicine) were grown in RPMI-1640 medium supplemented with 10% fetal bovine serum (FBS, HyClone) according to ATCC recommendations. Cells were washed with ice-cold FACS buffer (Dulbecco's PBS containing 2% FBS and 50 nM Dasatinib), counted, seeded at ∼50,000 viable cells per well in V-bottom 96-well plate for each mAb to be tested and incubated 60 min at 4 °C with serial tenfold dilutions of mAb in a total volume 100 μl per staining. Cells were washed with FACS buffer by centrifugation for 2 min at 800*g* followed by incubation with 1:500 dilution of secondary goat anti-human IgG PE Ab (SouthernBiotech) in FACS buffer. After washing, 5,000–10,000 live cell events were acquired using a three-laser LSR-II flow cytometer (BD Biosciences) and analysed with FlowJo software (Tree Star). The dead cell population was excluded using propidium iodide staining.

### Production and purification of recombinant RSV F protein and RSV mAbs

Plasmids encoding cDNAs for RSV subgroup A strain A2 F protein (wild-type post-fusion lacking the signal peptide and transmembrane domain), the SC-TM construct and the subgroup B strain 18537 protein construct (wild-type post-fusion F protein lacking the signal peptide and transmembrane domain) were synthesized (Genscript). The RSV B 18537 Ds-Cav1 (pre-fusion) construct was a gift from B. Graham (NIH). Plasmids were expanded in *E. coli* DH5α cells and DNA was purified using Qiagen Plasmid Maxiprep kits (Qiagen). For each litre of transfected culture, 1.3 mg of plasmid DNA was mixed with 2 mg of polyethylenimine in Opti-MEM I + GlutaMAX cell culture medium (Fisher). After 10 min, the DNA mixture was added to HEK293 cells at 1 × 10^6^ cells per ml. The culture supernatant was collected after 6 days and the protein was purified by a HiTrap Talon crude (GE Healthcare Life Sciences) column for RSV F protein variants and mutants. Expression and purification of mAbs 101F, motavizumab and D25 have been described previously^14^. Commercial preparations of palivizumab (Synagis; Medimmune) were obtained from the pharmacy at Vanderbilt University Medical Center.

### Crystallization and structure determination of the hRSV90-RSV F SC-TM complex

To crystallize hRSV90-Fab in complex with RSV A2 F SC-TM, both Fab cleaved from hybridoma-derived IgG of hRSV90 and RSV A2 F were buffer-exchanged in excess into 50 mM Tris pH 7.5, 50 mM NaCl. hRSV90-Fab was mixed in excess with RSV A2 F SC-TM protein and incubated at 37 °C for 2 h. Following this, the sample was subjected to size exclusion chromatography (S200, 16/600, GE Healthcare Life Sciences) in 50 mM Tris pH 7.5, 50 mM NaCl. The complex was concentrated to 10 mg ml^−1^ and crystals were obtained in Hampton Crystal Screen HT in various conditions. The best diffracting crystals were obtained in 30% PEG 400, 200 mM MgCl_2_·6H_2_O and 100 mM HEPES pH 7.5. X-ray diffraction data were collected at the Advanced Photon Source LS-CAT beamline 21-ID-G. Data were indexed and scaled using X-ray Detector Software^20^ and were significantly anisotropic. The data were submitted to the diffraction anisotropy server and the data were truncated to 3.1 Å along the c* axis and to 3.6 Å along the a*/b* axes. A molecular replacement solution was obtained in Phaser^21^ using the RSV A2 F SC-TM structure (PDB: 5C6B) and by separately searching the variable and constant regions of a poly-alanine truncated Fab structure (PDB: 4Q9Q). The structure of the complex was completed by manually building in COOT (ref. 22) followed by subsequent rounds of manual rebuilding and refinement in Phenix^21^. The data collection and refinement statistics are shown in Supplementary Table 1.

### RSV F mutant western blot

An SDS–PAGE gel (4–12% Bis-Tris) was run for the RSV F SC-TM protein mutants. The proteins were transferred to a PVDF membrane using the iBlot system (Thermo Fisher Scientific). The membrane was blocked in 5% nonfat milk for 1 h and then washed three times with PBS-T. Following this, the membrane was incubated with a 1:1,000 dilution of monoclonal anti-polyhistidine-alkaline phosphatase antibody (Sigma, #A5588) in 5% nonfat milk for 1 h. The membrane was washed three times with PBS-T and incubated with BM purple chromogenic substrate (Roche, 11442074001).

### Data availability

The structure of the hRSV90-RSV F SC-TM complex has been deposited in the Protein Data Bank under accession code 5TPN.

## Supplementary Material

Supplemental

## Figures and Tables

**Table 1:** Human antibody characterization by expression level, isotype, neutralization potency and binding affinity.

hRSV mAb	Average expression level (mg l^−1^)	IgG subclass	Light chain	Neutralization for indicated strain (IC_50_, ng ml^−1^)			F-protein binding for indicated strain (EC_50_, ng ml^−1^)			
	
				A2	18537 B	Long	RSV A2 SC-TM (pre)	RSV A2 (post)	RSV B 18537 Ds-Cav1 (pre)	RSV B 18537 (post)
90	26	1	κ	4	10	35	28	>	31	>
20	1.5	1	κ	45	11	3	33	>	45	>
130	6.7	2	κ	72	93	6	710	>	820	>
61	7	1	κ	90	164	21	27	>	26	>
7	11	1	λ	106	122	28	82	>	64	>
131	3.3	1	κ	115	147	20	53	370	46	92
75	30	1	λ	125	128	28	44	>	40	>
12	23	1	κ	176	126	46	28	285	27	>
141	1.9	1	κ	176	147	13	29	>	36	>
137	14	1	κ	210	58	29	27	>	22	>
106	5.5	1	λ	210	75	79	46	>	33	>
139	5.1	1	κ	323	400	33	40	>	41	>
97	4.7	1	κ	350	65	69	53	>	40	>
Control mAbs										
D25	–	1	κ	21	>	9	95	>	>	>
101F	–	1	κ	402	109	92	40	85	54	96
Motavizumab	–	1	κ	123	91	9	17	83	22	84
Palivizumab	–	1	κ	1,900	1,300	212	19	24	46	81
AM14	–	1	κ	14	58	43	106	>	>	198

The average expression level is the result from two 1 litre hybridoma cultures grown for three weeks. EC_50_ values correspond to the concentration at which the half-maximum signal was obtained in ELISA, based on the optical density at 405 nm. Neutralization values were determined using a plaque-reduction assay, where the IC_50_ corresponds to the mAb concentration at which 50% plaque reduction was observed. > indicates signal not detected below 1 μg ml^−1^. Each value is an average of three technical replicates for neutralization experiments and four technical replicates for binding data. Each experiment was repeated independently at least twice.

**Table 2:** Antibody gene usage for selected neutralizing mAbs.

hRSV mAb	Heavy chain					Light chain			
	
	Vh gene % identity	Jh gene % identity	D_H_ gene	HCDR lengths	Junction	Vl gene % identity	Jl gene % identity	LCDR lengths	Junction
12	*IGHV1*– 18*01 89%	IGHJ4*02 85%	IGHD6– 13*01	8.8.14	CARGPPVIAAVSLEYW	IGKV2– 30*02 96%	IGKJ2*04 95%	11.3.9	CMQATHWPGSF
131	*IGHV1*– 18*01 93%	IGHJ4*02 87%	IGHD6– 13*01	8.8.15	CAREPPSLTAAGLLDYW	IGKV2– 30*01 82%	IGKJ4*01 97%	11.3.9	CQQYNNWPLTF
61	IGHV1– 18*01 93%	IGHJ4*02 83%	IGHD2– 15*01	8.8.13	CARDNGVVVGPPDYW	IGKV1– 5*03 96%	IGKJ1*01 94%	6.3.8	CQYYHSLSAF
139	IGHV1– 18*01 92%	IGHJ1*01 84%	IGHD3– 10*02	8.8.14	CSRQSGVSGVPEFQDW	IGKV2– 30*01 96%	IGKJ5*01 95%	11.3.10	CMQGTHWPPPTF
90	*IGHV3*– 9*01 97%	IGHJ6*02 89%	IGHD3– 10*01	8.8.18	CVRDAYVSGSDYYYYGLDVW	IGKV3– 15*01 96%	IGKJ4*01 83%	6.3.9	CQQYNNWPLTF
20	*IGHV3*– 9*01 97%	IGHJ6*02 92%	IGHD3– 10*01	8.8.21	CVKDNYASGSYSSYYYYYGLDLW	IGKV3– 15*01 96%	IGKJ5*01 100%	6.3.9	CQQYNNWPITF
130	IGHV3– 9*01 92%	IGHJ6*02 77%	IGHD3– 22*01	8.8.19	CVKDSHYFDNSGSYTYGLDVW	IGKV3– 15*01 94%	IGKJ4*01 97%	6.3.9	CQQYNNWPLTF
97	*IGHV3*– 9*01 91%	IGHJ4*02 85%	IGHD6– 19*01	8.8.17	CGKDVFWAVAGTGGPIDSW	IGKV1– 27*01 95%	IGKJ2*04 84%	6.3.10	CQNYNSAQMCSF

mAbs are grouped based on heavy chain V gene analysis. hRSV90, hRSV20, hRSV130 and hRSV97 use similar Vh genes, and hRSV12, hRSV131, hRSV61 and hRSV139 use similar Vh genes. hRSV90 and hRSV20 have nearly identical gene usage and hRSV20 has an HCDR3 insertion. Analysis was carried out by IMGT/VQUEST (ref. 18). The per cent identity for each predicted gene usage based on IMGT/VQUEST predictions is also displayed for Vh and Jh or Vl and Jl genes. Vh, heavy chain variable region gene; Jh, heavy chain joining region gene; Vl, light chain variable region gene; Jl, light chain joining region gene.

**Table 3:** Binding characteristics for hRSV mAbs to variant F proteins mutated at residues that contact mAb hRSV90.

hRSV mAb	Binding to indicated recombinant RSV F point mutant variant protein for residues contacting the indicated CDR of hRSV90 (EC_50_, ng ml^−1^)													

	HCDR1		HCDR2		HCDR3						LCDR1/2		LCDR3	
				
	K201A	K201R	S169A	S169R	S173A	S173R	D194A	D194R	N175A	N175R	D263A	D263R	T174A	T174R
90	21	18	23	19	10	>	42	32	28	11	22	18	18	>
20	20	13	15	14	365	>	20	19	50	20	16	18	16	>
75	19	15	14	16	16	17	26	22	66	20	15	23	7	12
12	25	17	24	26	29	47	33	30	24	>	17	>	23	27
130	280	160	195	201	260	>	302	344	480	>	1,074	250	270	>
61	43	47	59	65	62	98	144	105	72	48	49	>	41	>
137	31	26	34	77	29	37	99	>	22	>	29	>	40	>
106	55	45	48	50	51	83	134	197	82	116	46	124	48	39
139	34	31	37	40	43	75	54	78	56	>	36	>	41	164
141	32	30	39	196	36	41	140	>	>	>	44	>	271	>
131	21	15	18	18	21	18	27	28	68	22	19	23	19	14
7	92	62	79	95	71	186	748	695	158	255	62	163	86	64
97	84	65	63	62	46	173	178	286	106	106	42	145	55	37
Control mAbs													
D25	630	21	19	21	35	21	107	44	690	22	19	27	21	16
AM14	28	23	18	26	170	1	>	58	>	>	27	26	28	18

hRSV90 CDR loops interacting with specified RSV F SC-TM amino acid residues in the X-ray structure are indicated above each mutation. Both alanine and arginine mutations were tested at each of the seven positions. Each value is an average of four technical replicates. Each experiment was repeated independently at least twice. > indicates no signal was observed below 0.7 μg ml^−1^.
