# Supplementary material for: A novel pre-fusion conformation-specific neutralizing epitope on the respiratory syncytial virus fusion protein
Source: Nat Microbiol. Author manuscript; Available in PMC 2017 Jun 8. (PMC5463187; doi:10.1038/nmicrobiol.2016.271)
Supplement: Supplemental [file NIHMS862890-supplement-Supplemental.pdf]

nature microbiology

SUPPLEMENTARY INFORMATION

VOLUME: 2 | ARTICLE NUMBER: 16271

In the format provided by the authors and unedited.

A novel pre-fusion conformation-specific neutralizing epitope on the respiratory syncytial virus fusion protein

Jarrod J. Mousa, Nurgun Kose, Pranathi Matta, Pavlo Gilchuk and James E. Crowe Jr

© 2017 Macmillan Publishers Limited, part of Springer Nature. All rights reserved.

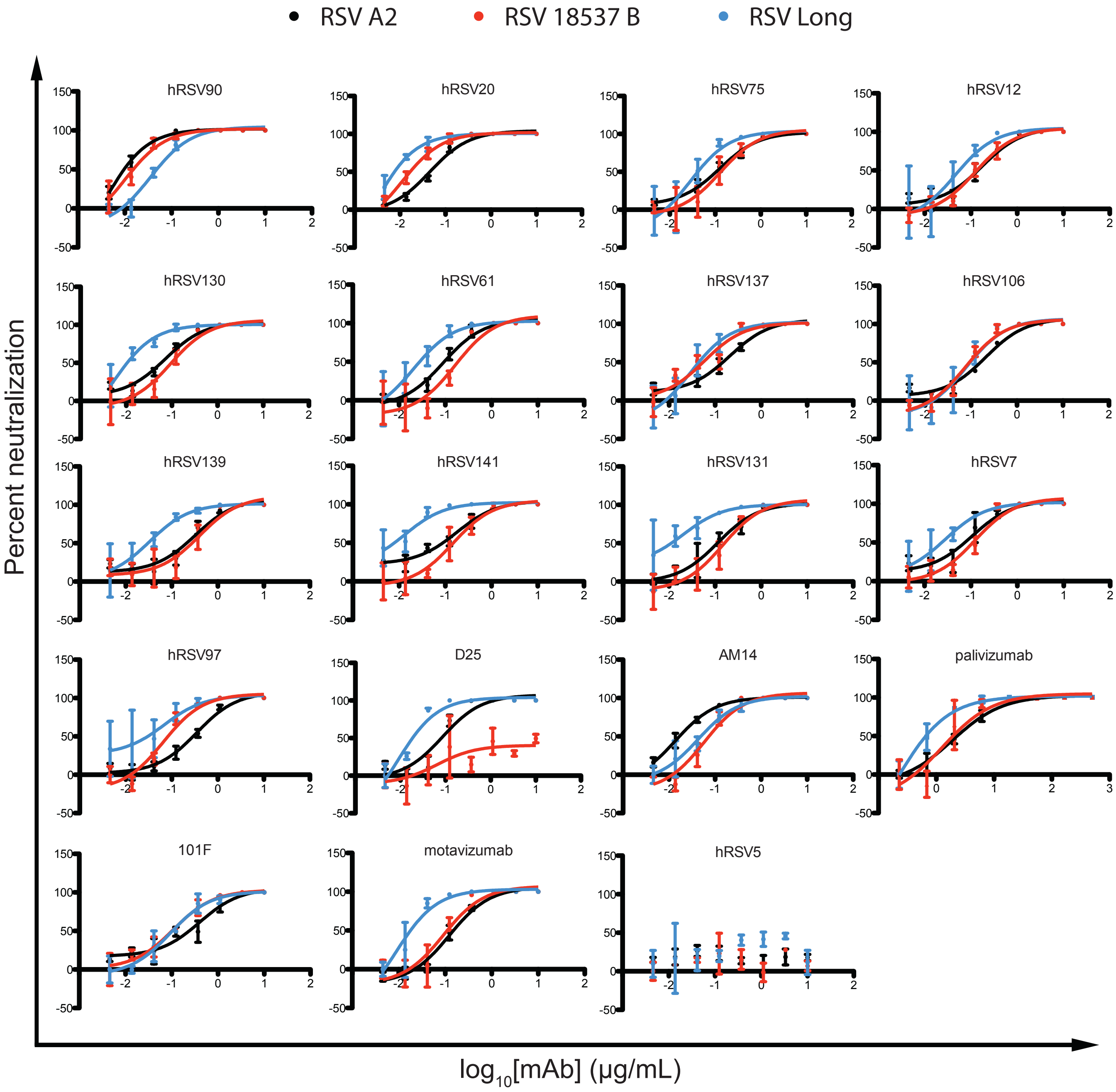

Supplementary Figure 1. Neutralization curves for the newly isolated RSV F-specific mAbs. IC_{50} values are displayed in Table 1. Error bars represent the standard deviation of three technical replicates from one of at least two independent experiments. A non-neutralizing RSV F specific mAb, hRSV5, was used as a negative control. MAbs D25, 101F, AM14, motavizumab, and palivizumab were included as positive controls.

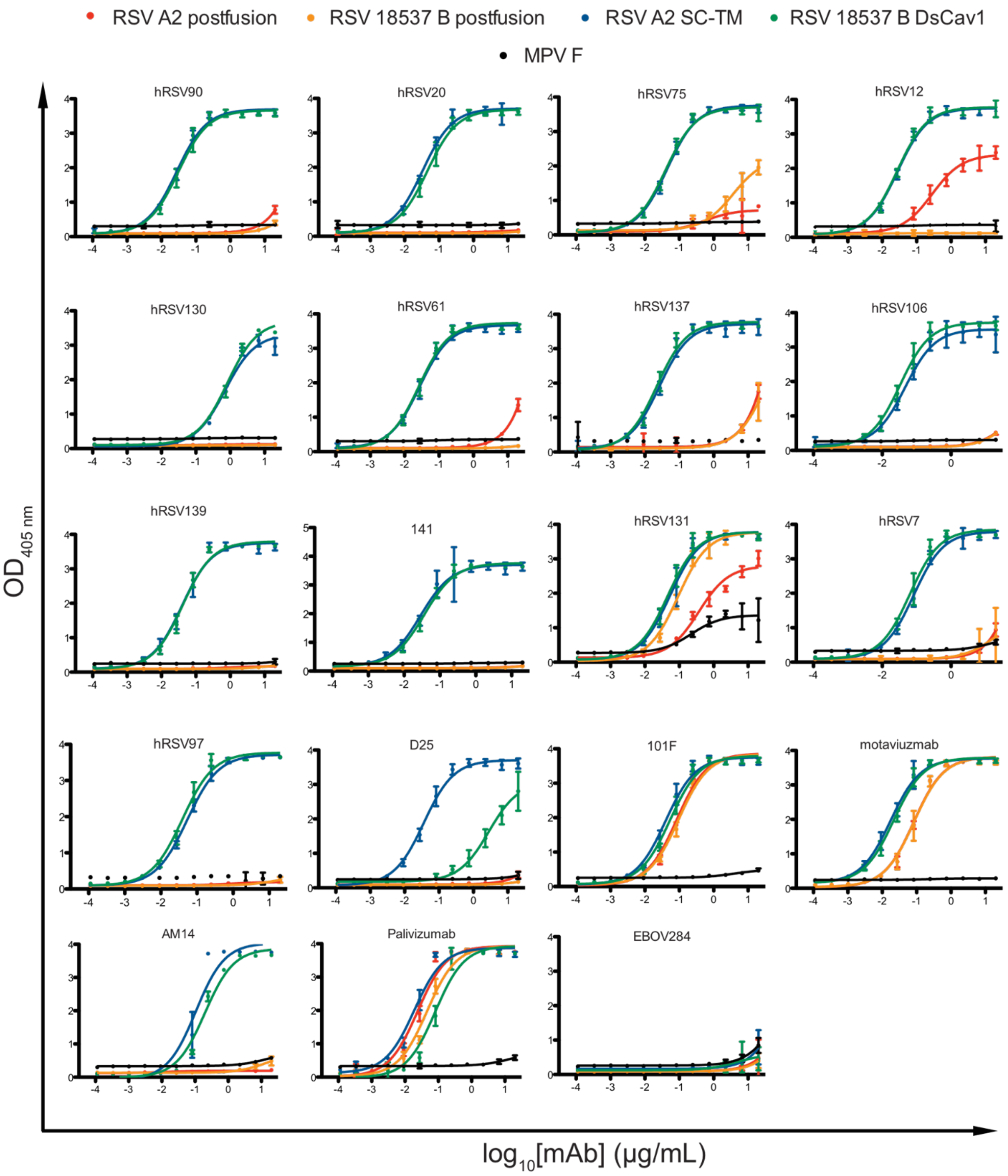

Supplementary Figure 2. ELISA binding curves for the newly isolated mAbs and positive controls to RSV F protein strain and construct variants. The metapneumovirus F protein was used as a negative binding control. An Ebola-virus specific mAb EBOV284 was used as a negative mAb control. Error bars indicate 95% confidence intervals of four technical replicates from one of at least two independent experiments. EC_{50} values for these curves are displayed in Table 1.

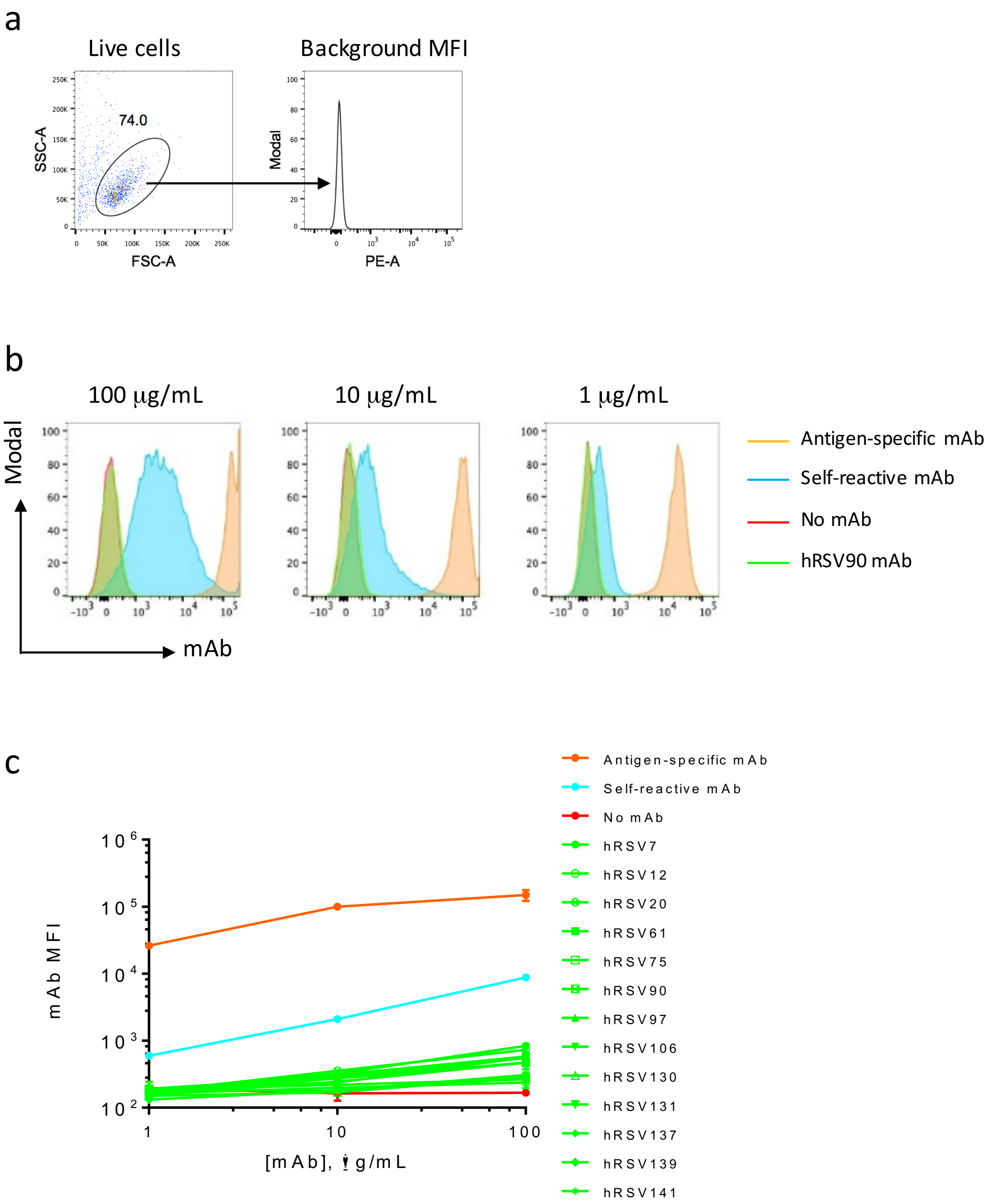

Supplementary Figure 3. **Assessing self-reactivity of hRSV mAbs by flow cytometry.** Jurkat cell line was stained with individual mAbs followed by incubation with secondary phycoerythrin (PE)-conjugated Ab and flow cytometric analysis. (a) Gating strategy for measuring binding of mAb to Jurkat cells. (b) Representative flow cytometric histograms showing dose-dependent binding of antigen-specific, self-reactive, or hRSV90 mAbs to Jurkat cells. Binding of BDBV289 Ebola virus GP-specific mAb to transfected Jurkat cells that express Ebola virus GP on their surface served as positive control for antigen-specific mAb (orange histogram); a mAb with known self-reactivity (BDBV223) served as a control for self-reactivity (light blue histogram); Jurkat stained with secondary detection PE-conjugated Ab only served as a control for assay background (red histogram). (c) Dose-dependent binding of hRSV mAbs to Jurkat cells measured as mean fluorescence intensity (MFI). Error bars represent the SD of two technical replicates.

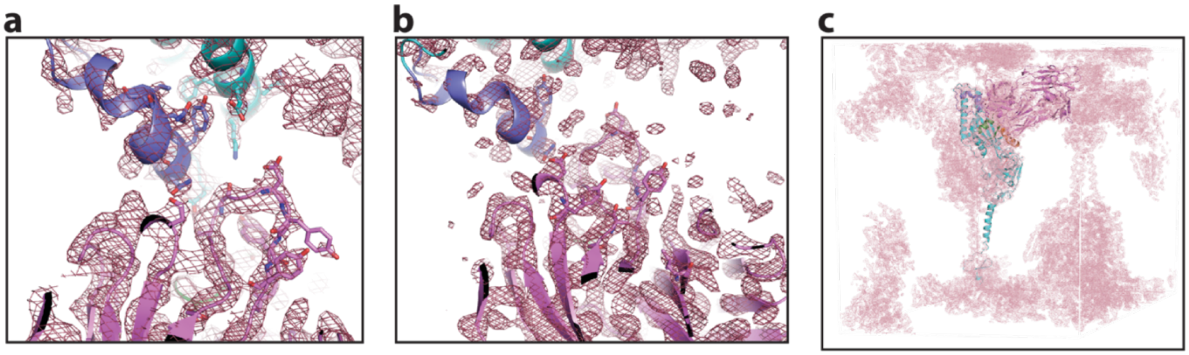

Supplementary Figure 4. Density maps for the hRSV90-RSV F A2 SC-TM interface. (a) $2F_o$-$2F_c$ density map. (b) Simulated annealing composite omit density map of the same interface. (c) $2F_o$-$2F_c$ Density map of the entire structure showing density for the foldon trimerization domain.

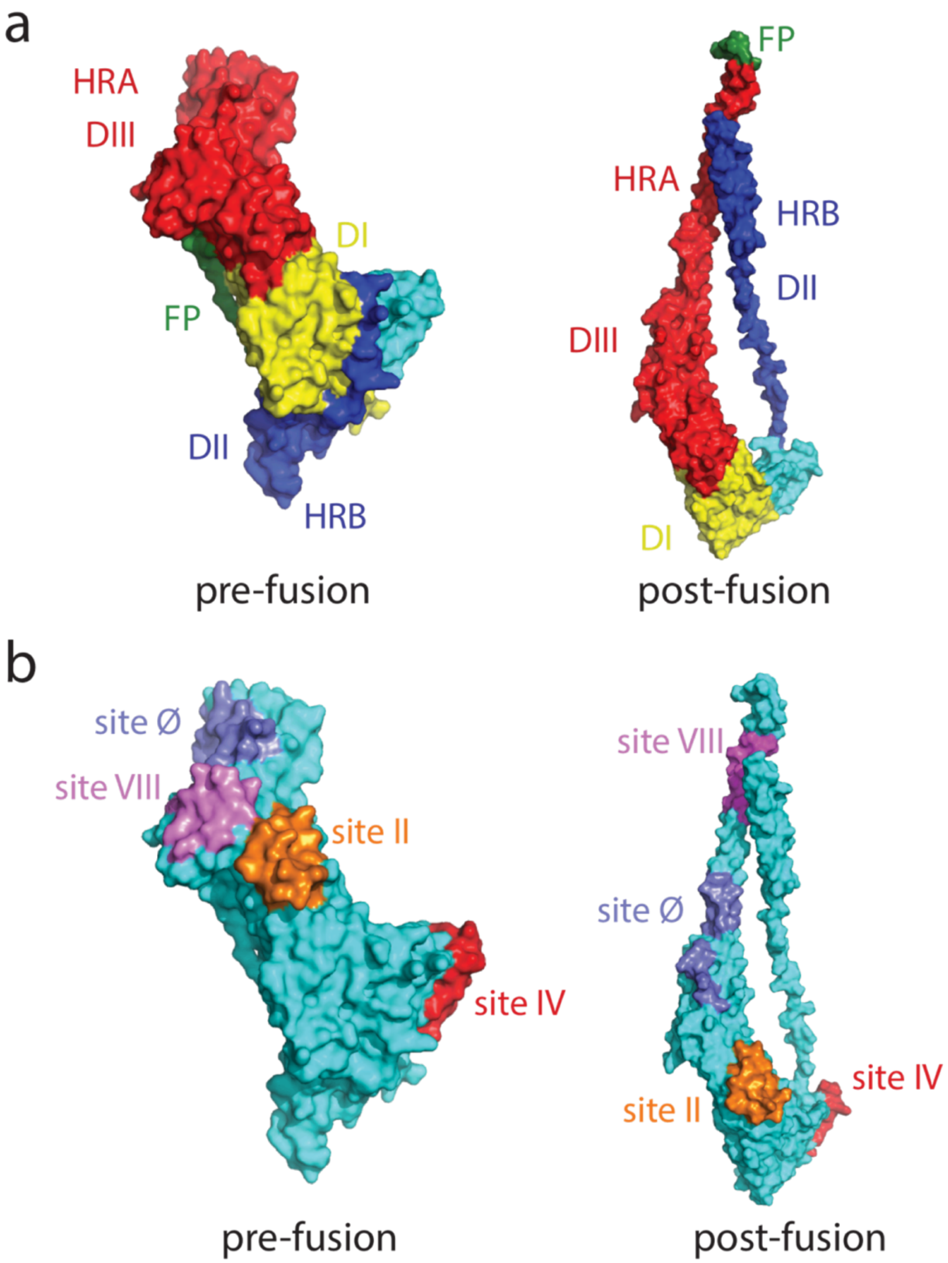

Supplementary Figure 5. Structural and antigenic regions of the RSV F protein. (a) Structural regions of the RSV F protein are shown with corresponding labels in both pre-fusion and post-fusion structures. HRA is heptad repeat A (red as part of DIII), HRB is heptad repeat B (blue as part of DII), FP is fusion peptide (green), and DI is shown in yellow. (b) Antigenic regions in the pre-fusion and post-fusion RSV F structures are colored. Site IV is red, site II is orange, site Ø is blue, and site VIII is magenta. Residues comprising antigenic sites Ø and VIII are rearranged in the post-fusion conformation, resulting in loss of mAb binding. Site VIII becomes part of the six-helix bundle of the post-fusion RSV F protein.

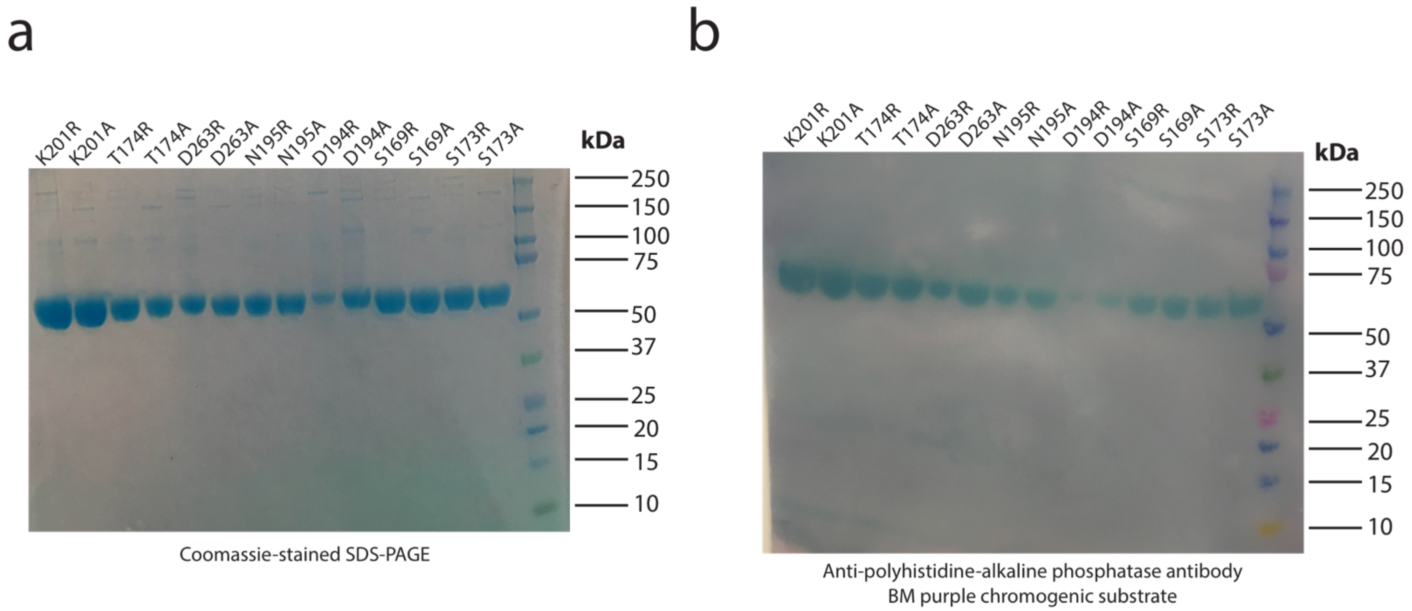

Supplementary Figure 6. RSV F SC-TM mutants. (a) A Coomassie-stained SDS-PAGE is displayed with bands displayed for each purified mutant protein. (b) A corresponding western blot is shown using a monoclonal anti-polyhistidine-alkaline phosphatase antibody with BM purple chromogenic substrate to visualize the RSV F mutants directly on the PVDF membrane. Purifications, SDS-PAGE, and western blot were conducted once.

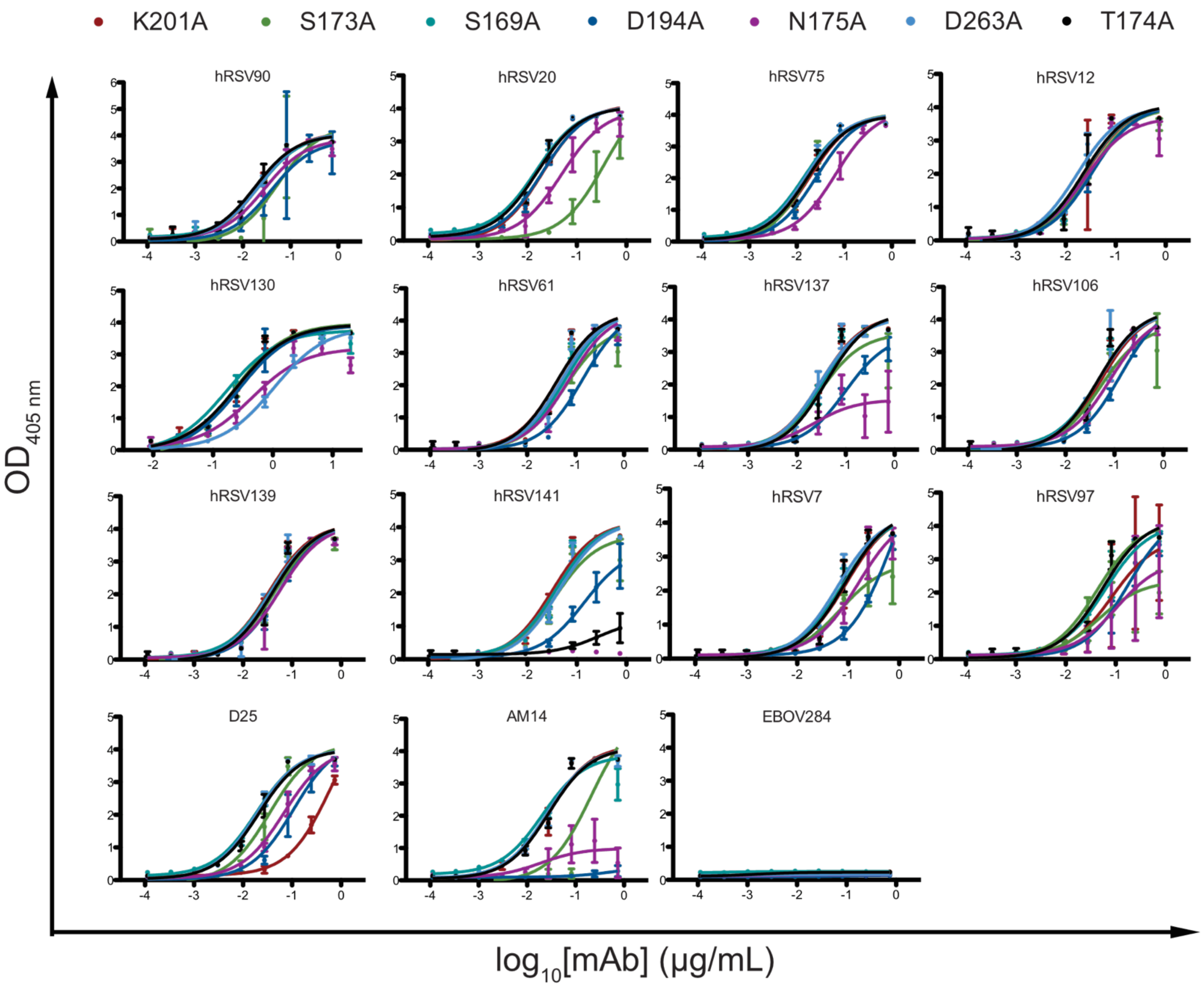

Supplementary Figure 7. ELISA binding curves for RSV F SC-TM alanine mutations at the hRSV90 binding site. Error bars indicate 95% confidence intervals of four technical replicates from one of at least two independent experiments.

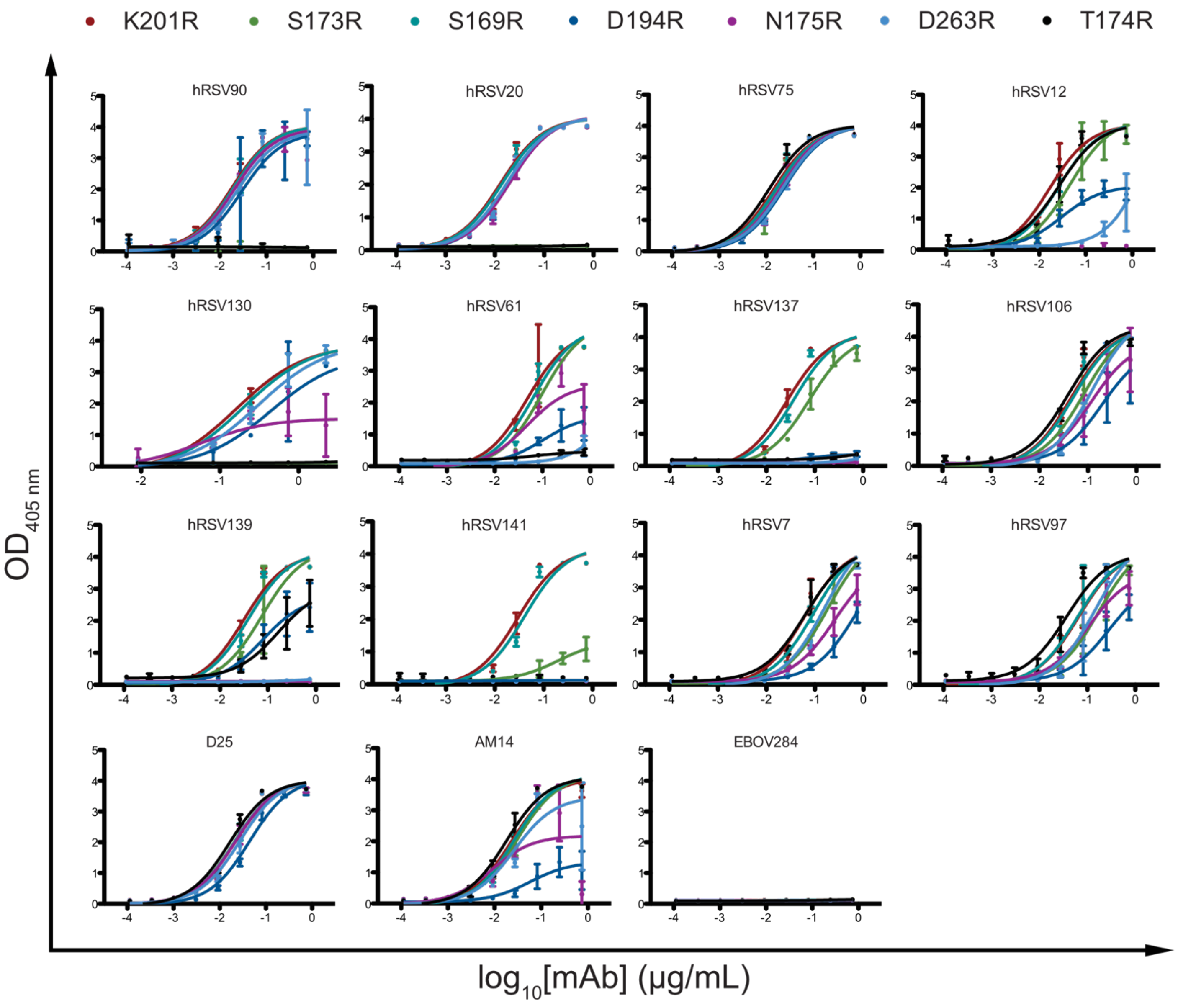

Supplementary Figure 8. ELISA binding curves for RSV F SC-TM arginine mutations at the hRSV90 binding site. Error bars indicate 95% confidence intervals of four technical replicates from one of at least two independent experiments.

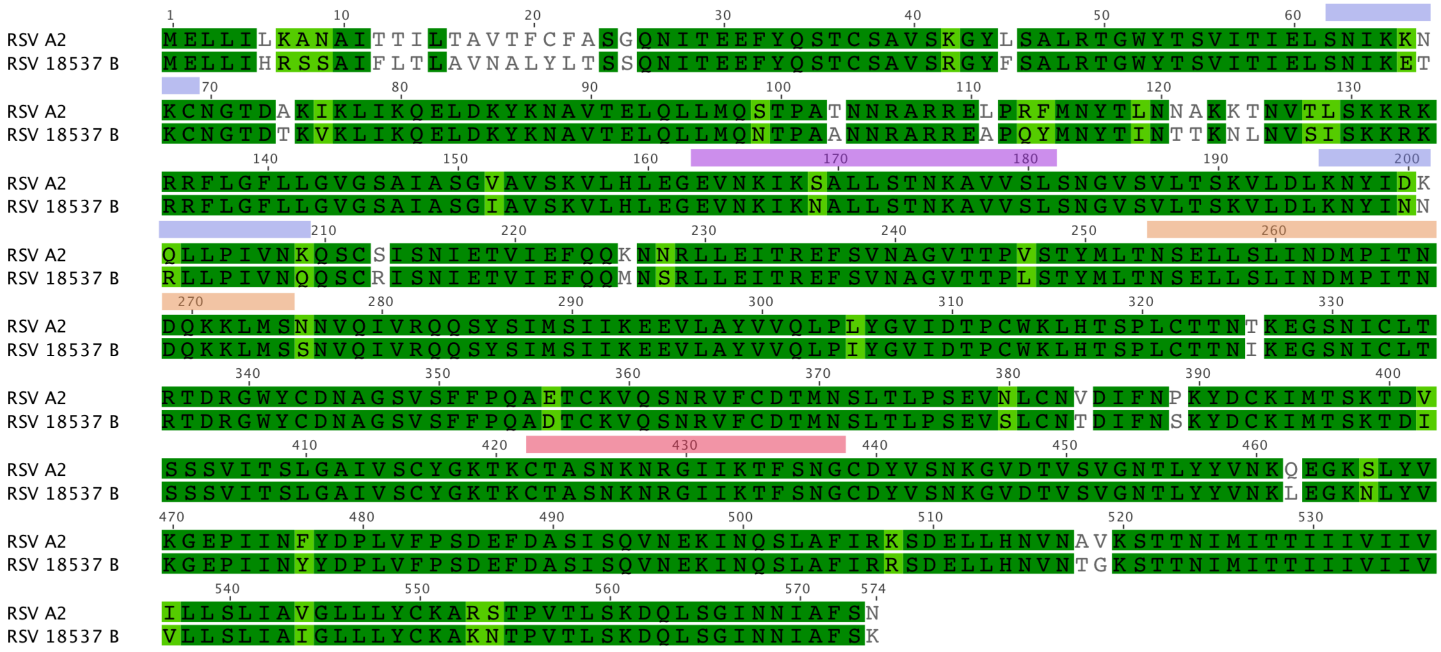

Supplementary Figure 9. Alignment of RSV F proteins from subgroups A and B. Amino acids in green are conserved among the two proteins, those in light green are semi-conserved, and those in white are not conserved. Antigenic sites are shaded above the corresponding sequences, with site Ø in blue, site VIII in magenta, site II in orange, and site IV in red.

Supplementary Table 1. Data collection and refinement statistics

**Data collection***	hRSV90+RSV A2 F SC-TM	
Beamline	LS-CAT 21-ID-G	Anisotropy correction
Number of crystals	1	
Space group	R 3 2 H	
Cell dimensions		
a, *b*, *c* (Å)	148.2, 148.2, 538.2	
α, β, γ (°)	90, 90, 120	
Resolution (Å)	49.23 – 3.14 (3.26 – 3.14)	a= 3.6, b= 3.6, c= 3.1
R_{merge}	0.385 (4.715)	0.266 (0.768)
$I / \sigma I$	5.2 (0.5)	6.7 (2.2)
Completeness (%)	99.9 (99.8)	75.2 (4.1)
Redundancy	5.9 (5.9)	4.4 (0.2)
**Refinement**		
Resolution (Å)		48.31 – 3.14
No. unique reflections		30527 (272)
R_{work} / R_{free}		0.2212 (0.2603)
No. atoms		
Protein		7077
B-factors		
Protein		71.68
R.m.s. deviations		
Bond lengths (Å)		0.012
Bond angles (°)		1.41
Ramachandran statistics		
Favored regions (%)		95
Allowed regions (%)		4.8
Outliers (%)		0.22

Values in parentheses are for the highest resolution data shell.
